# Tetra­aqua­(2,2′-bipyridine-5,5′-dicarboxyl­ato-κ^2^ *N*,*N*′)nickel(II) dihydrate

**DOI:** 10.1107/S1600536809035910

**Published:** 2009-09-12

**Authors:** Hua Yang

**Affiliations:** a Department of Chemistry, Mudanjiang Teachers College, Mudanjiang 157012, People’s Republic of China

## Abstract

In the title compound, [Ni(C_12_H_6_N_2_O_4_)(H_2_O)_4_]·2H_2_O, obtained from a basic solution of 2,2′-bipyridine-5,5′-dicarboxyl­ate and nickel(II) chloride in water, the central Ni(II) cation (site symmetry 2) is coordinated by two N atoms from the 2,2′-bipyridine-5,5′-dicarboxyl­ate ligand and four aqua O atoms. The N—Ni—N angle is 78.64 (8)°. Weak but significant π–π stacking inter­actions exist between the pyridine rings with a centroid–centroid distance of 3.652 (8) Å. In addition, four O atoms of the two carboxyl groups form hydrogen bonds with both coordinated and uncoordinated water mol­ecules, forming an infinite three-dimensional network.

## Related literature

For attempts to synthesize 5,5′- and 6,6′-substituted 2,2′-bipyridine derivatives, see: He *et al.* (2009 ▶); Karaca *et al.* (2009 ▶); Yousefi *et al.* (2008 ▶).
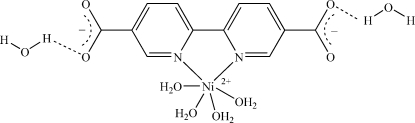


## Experimental

### 

#### Crystal data

[Ni(C_12_H_6_N_2_O_4_)(H_2_O)_4_]·2H_2_O
*M* *_r_* = 408.97
Monoclinic, 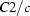

*a* = 12.4787 (2) Å
*b* = 9.8152 (2) Å
*c* = 12.6533 (2) Å
β = 92.107 (2)°
*V* = 1548.74 (5) Å^3^
*Z* = 4
Mo *K*α radiation
μ = 1.31 mm^−1^
*T* = 120 K
0.18 × 0.16 × 0.10 mm

#### Data collection

Bruker APEXII CCD area-detector diffractometer
Absorption correction: multi-scan (*SADABS*; Bruker, 2005 ▶) *T*_min_ = 0.730, *T*_max_ = 0.828
8276 measured reflections
1691 independent reflections
1379 reflections with *I* > 2σ(*I*)
*R*_int_ = 0.037

#### Refinement

*R*[*F* ^2^ > 2σ(*F* ^2^)] = 0.025
*wR*(*F* ^2^) = 0.065
*S* = 0.98
1691 reflections
138 parameters
6 restraints
H atoms treated by a mixture of independent and constrained refinement
Δρ_max_ = 0.44 e Å^−3^
Δρ_min_ = −0.33 e Å^−3^

### 

Data collection: *APEX2* (Bruker, 2005 ▶); cell refinement: *SAINT* (Bruker, 2005 ▶); data reduction: *SAINT*; program(s) used to solve structure: *SHELXS97* (Sheldrick, 2008 ▶); program(s) used to refine structure: *SHELXL97* (Sheldrick, 2008 ▶); molecular graphics: *ORTEP-3 for Windows* (Farrugia, 1997 ▶); software used to prepare material for publication: *WinGX* (Farrugia, 1999 ▶).

## Supplementary Material

Crystal structure: contains datablocks I, global. DOI: 10.1107/S1600536809035910/ng2636sup1.cif

Structure factors: contains datablocks I. DOI: 10.1107/S1600536809035910/ng2636Isup2.hkl

Additional supplementary materials:  crystallographic information; 3D view; checkCIF report

## Figures and Tables

**Table 1:** Hydrogen-bond geometry (Å, °)

*D*—H⋯*A*	*D*—H	H⋯*A*	*D*⋯*A*	*D*—H⋯*A*
O5—H52⋯O3^i^	0.837 (10)	2.178 (16)	2.9566 (19)	155 (3)
O2—H22⋯O3^ii^	0.834 (10)	1.921 (12)	2.7410 (18)	168 (3)
O5—H51⋯O4^iii^	0.834 (10)	1.913 (11)	2.7286 (19)	166 (2)
O2—H21⋯O4^iv^	0.833 (10)	1.852 (10)	2.6831 (17)	175 (2)
O1—H11⋯O4^iv^	0.843 (9)	2.650 (17)	3.1740 (18)	121.6 (16)
O1—H11⋯O3^iv^	0.843 (9)	2.097 (10)	2.9386 (18)	175.9 (19)
O1—H12⋯O5	0.834 (10)	1.861 (11)	2.688 (2)	171 (3)

Symmetry codes: (i) 
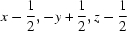
; (ii) 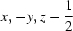; (iii) 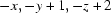; (iv) 
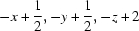
.

**Fig. 1.**
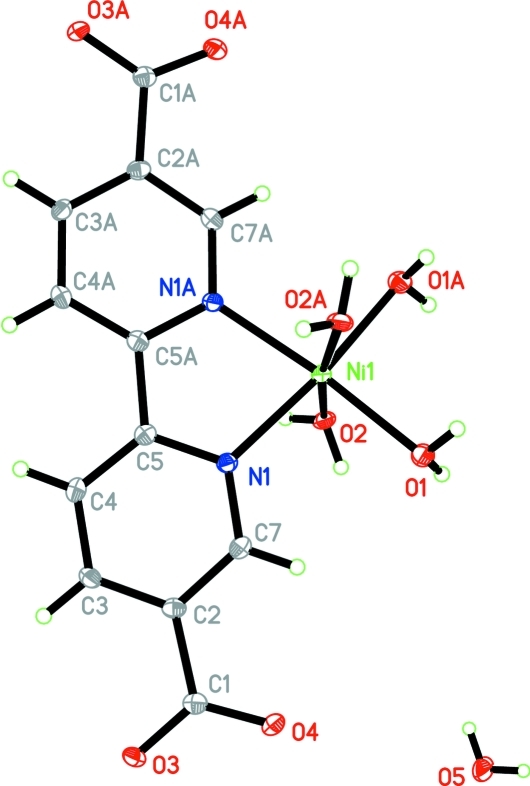
The molecular structure with atom labels and 30% probability displacement ellipsoids for non-H atoms.

**Fig. 2.**
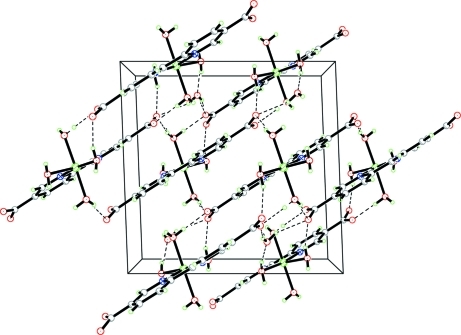
The packing diagram of molecules, viewed down the b axis, with the weak interactions shown as dashed lines.

## supplementary crystallographic information

### Comment

2,2'-Bipyridine was used very commonly as ending complexing ligand. Great efforts have been made to synthesize 5,5' and 6,6' position substituted derivatives (He *et al.*, 2009; Karaca *et al.*, 2009; Yousefi *et al.*, 2008). Here we reported the crystal structure of a complexing compound of Ni(II) coordinating to 4,4'dicarboxyl substituted 2,2'-bipyridine derivative.

The central Ni cation coordinated to two N atoms from dianions of one 2,2'-bipyridine-5,5'-dicarboxylate and four O atoms from four waters, forming a distorted octahedral system. Ni(II) cation lies on the twofold axis of the crystal lattice. Two Ni—N bonds were generated by *C*2 symmetry operation from each other with bond length 2.0706 (14). Four Ni—O bond lengths are nearly equal, two of which 2.0610 (12), and another two 2.0801 (13). Each of two equivalent carboxyl anions has two unequivalent oxygen atoms, C—O bond lengths of which are equalized to be 1.266 (2) and 1.254 (2), respectively. All O atoms of carboxyls formed hydrogen bonds with both complexed waters from another complexing supermolecule and free waters into three dimentional infinite hydrgon bonding network, which stablized the whole crystal structure, along with pi-pi stacking of aromatic pyridine rings.

### Experimental

A solution of 2,2'-bipyridine-5,5'-dicarboxylate (23.2 mg, 0.1 mmol) and NiCl_2_.6H_2_O (23.8 mg, 0.1 mmol) was added an aqueous solution of NaOH (0.1 mmol/ml) to adjust pH as 7.0–7.5 at room temperature. A small amount of white precipitate was removed from the resulting solution. Prism colorless crystals were obtained by slow evaporation at room temperature over a period of 10 days.

### Refinement

All H atoms bonded to O atoms of ligand water and free water molecules were located in a difference map, and the distances of the O–H bonds were fixed to 0.82 Å. The other H atoms were placed in calculated positions and refined as riding, with C–H = 0.93 Å, and *U*_iso_(H) = 1.2 *U*_eq_(C).

### Crystal data

**Table:**

[Ni(C_12_H_6_N_2_O_4_)(H_2_O)_4_]·2H_2_O	*F*(000) = 848.0
*M**_r_* = 408.97	*D*_x_ = 1.754 Mg m^−^^3^
Monoclinic, *C*2/*c*	Mo *K*α radiation, λ = 0.71073 Å
Hall symbol: -C 2yc	Cell parameters from 8276 reflections
*a* = 12.4787 (2) Å	θ = 3.1–27.0°
*b* = 9.8152 (2) Å	µ = 1.31 mm^−^^1^
*c* = 12.6533 (2) Å	*T* = 120 K
β = 92.107 (2)°	Prism, colourless
*V* = 1548.74 (5) Å^3^	0.18 × 0.16 × 0.10 mm
*Z* = 4	

### Data collection

**Table:**

Bruker APEXII CCD area-detector diffractometer	1691 independent reflections
Radiation source: fine-focus sealed tube	1379 reflections with *I* > 2σ(*I*)
graphite	*R*_int_ = 0.037
φ and ω scans	θ_max_ = 27.0°, θ_min_ = 3.1°
Absorption correction: multi-scan (*SADABS*; Bruker, 2005)	*h* = −15→15
*T*_min_ = 0.730, *T*_max_ = 0.828	*k* = −12→12
8276 measured reflections	*l* = −14→16

### Refinement

**Table:**

Refinement on *F*^2^	Primary atom site location: structure-invariant direct methods
Least-squares matrix: full	Secondary atom site location: difference Fourier map
*R*[*F*^2^ > 2σ(*F*^2^)] = 0.025	Hydrogen site location: inferred from neighbouring sites
*wR*(*F*^2^) = 0.065	H atoms treated by a mixture of independent and constrained refinement
*S* = 0.98	*w* = 1/[σ^2^(*F*_o_^2^) + (0.0426*P*)^2^] where *P* = (*F*_o_^2^ + 2*F*_c_^2^)/3
1691 reflections	(Δ/σ)_max_ = 0.001
138 parameters	Δρ_max_ = 0.44 e Å^−^^3^
6 restraints	Δρ_min_ = −0.33 e Å^−^^3^

### Special details

**Table:**

Geometry. All e.s.d.'s (except the e.s.d. in the dihedral angle between two l.s. planes) are estimated using the full covariance matrix. The cell e.s.d.'s are taken into account individually in the estimation of e.s.d.'s in distances, angles and torsion angles; correlations between e.s.d.'s in cell parameters are only used when they are defined by crystal symmetry. An approximate (isotropic) treatment of cell e.s.d.'s is used for estimating e.s.d.'s involving l.s. planes.
Refinement. Refinement of *F*^2^ against ALL reflections. The weighted *R*-factor *wR* and goodness of fit *S* are based on *F*^2^, conventional *R*-factors *R* are based on *F*, with *F* set to zero for negative *F*^2^. The threshold expression of *F*^2^ > σ(*F*^2^) is used only for calculating *R*-factors(gt) *etc*. and is not relevant to the choice of reflections for refinement. *R*-factors based on *F*^2^ are statistically about twice as large as those based on *F*, and *R*- factors based on ALL data will be even larger.

### Fractional atomic coordinates and isotropic or equivalent isotropic displacement parameters (Å^2^)

**Table:**

	*x*	*y*	*z*	*U*_iso_*/*U*_eq_	
Ni1	0.0000	0.27554 (3)	0.7500	0.01072 (11)	
O1	0.03285 (11)	0.42083 (13)	0.86619 (11)	0.0142 (3)	
H11	0.0898 (11)	0.4670 (18)	0.8664 (17)	0.019 (6)*	
H12	−0.0189 (14)	0.475 (2)	0.864 (2)	0.044 (8)*	
O2	0.15591 (10)	0.29226 (13)	0.70290 (10)	0.0129 (3)	
H21	0.1953 (16)	0.315 (2)	0.7545 (13)	0.032 (7)*	
H22	0.184 (2)	0.2274 (19)	0.672 (2)	0.052 (9)*	
O3	0.26260 (10)	−0.06943 (12)	1.12935 (10)	0.0145 (3)	
O4	0.21890 (10)	0.15053 (12)	1.12590 (10)	0.0166 (3)	
O5	−0.12732 (13)	0.60197 (15)	0.83875 (13)	0.0301 (4)	
H51	−0.1494 (18)	0.6770 (14)	0.8598 (18)	0.033 (7)*	
H52	−0.1726 (19)	0.580 (3)	0.7913 (18)	0.067 (10)*	
N1	0.04499 (11)	0.11232 (14)	0.84534 (11)	0.0106 (3)	
C1	0.21332 (13)	0.03194 (17)	1.08927 (14)	0.0119 (4)	
C2	0.14678 (14)	0.01069 (17)	0.98899 (14)	0.0116 (4)	
C3	0.13045 (14)	−0.11715 (17)	0.94398 (14)	0.0119 (4)	
H3	0.1595	−0.1960	0.9778	0.014*	
C4	0.07152 (14)	−0.12874 (17)	0.84950 (14)	0.0126 (4)	
H4	0.0596	−0.2156	0.8180	0.015*	
C5	0.03040 (13)	−0.01275 (17)	0.80161 (14)	0.0108 (4)	
C7	0.10106 (14)	0.12224 (17)	0.93712 (14)	0.0117 (4)	
H7	0.1100	0.2097	0.9683	0.014*	

### Atomic displacement parameters (Å^2^)

**Table:**

	*U*^11^	*U*^22^	*U*^33^	*U*^12^	*U*^13^	*U*^23^
Ni1	0.01160 (18)	0.00978 (17)	0.01056 (18)	0.000	−0.00259 (12)	0.000
O1	0.0113 (7)	0.0133 (7)	0.0177 (7)	0.0017 (5)	−0.0029 (5)	−0.0029 (5)
O2	0.0122 (6)	0.0148 (7)	0.0115 (7)	0.0004 (5)	−0.0030 (5)	−0.0024 (5)
O3	0.0155 (7)	0.0126 (6)	0.0151 (7)	−0.0005 (5)	−0.0059 (5)	0.0034 (5)
O4	0.0203 (7)	0.0133 (7)	0.0156 (7)	0.0013 (5)	−0.0064 (6)	−0.0043 (5)
O5	0.0286 (9)	0.0212 (8)	0.0392 (10)	0.0123 (6)	−0.0167 (7)	−0.0135 (7)
N1	0.0109 (8)	0.0106 (8)	0.0103 (8)	−0.0013 (5)	−0.0011 (6)	0.0005 (6)
C1	0.0101 (9)	0.0143 (9)	0.0113 (9)	−0.0019 (7)	0.0008 (7)	0.0014 (7)
C2	0.0108 (8)	0.0148 (9)	0.0092 (9)	−0.0002 (7)	0.0003 (7)	0.0005 (7)
C3	0.0110 (9)	0.0118 (9)	0.0128 (9)	0.0010 (6)	−0.0007 (7)	0.0020 (7)
C4	0.0132 (9)	0.0108 (9)	0.0140 (9)	−0.0006 (7)	0.0011 (7)	−0.0018 (7)
C5	0.0094 (8)	0.0124 (9)	0.0106 (9)	−0.0006 (6)	0.0009 (7)	−0.0007 (7)
C7	0.0110 (9)	0.0124 (9)	0.0118 (9)	−0.0014 (7)	0.0013 (7)	−0.0015 (7)

### Geometric parameters (Å, °)

**Table:**

Ni1—O2^i^	2.0622 (12)	O5—H52	0.837 (10)
Ni1—O2	2.0622 (12)	N1—C7	1.337 (2)
Ni1—N1^i^	2.0706 (14)	N1—C5	1.356 (2)
Ni1—N1	2.0706 (14)	C1—C2	1.505 (2)
Ni1—O1	2.0782 (13)	C2—C7	1.388 (2)
Ni1—O1^i^	2.0782 (13)	C2—C3	1.390 (2)
O1—H11	0.843 (9)	C3—C4	1.385 (2)
O1—H12	0.834 (10)	C3—H3	0.9500
O2—H21	0.833 (10)	C4—C5	1.380 (2)
O2—H22	0.834 (10)	C4—H4	0.9500
O3—C1	1.266 (2)	C5—C5^i^	1.486 (3)
O4—C1	1.254 (2)	C7—H7	0.9500
O5—H51	0.834 (10)		
			
O2^i^—Ni1—O2	170.87 (7)	C7—N1—C5	118.59 (14)
O2^i^—Ni1—N1^i^	89.51 (5)	C7—N1—Ni1	124.87 (11)
O2—Ni1—N1^i^	97.57 (5)	C5—N1—Ni1	115.66 (12)
O2^i^—Ni1—N1	97.57 (5)	O4—C1—O3	124.20 (16)
O2—Ni1—N1	89.51 (5)	O4—C1—C2	117.49 (15)
N1^i^—Ni1—N1	78.63 (8)	O3—C1—C2	118.27 (15)
O2^i^—Ni1—O1	84.53 (5)	C7—C2—C3	117.82 (16)
O2—Ni1—O1	89.21 (5)	C7—C2—C1	119.58 (15)
N1^i^—Ni1—O1	170.17 (6)	C3—C2—C1	122.59 (15)
N1—Ni1—O1	94.39 (5)	C4—C3—C2	119.54 (15)
O2^i^—Ni1—O1^i^	89.21 (5)	C4—C3—H3	120.2
O2—Ni1—O1^i^	84.53 (5)	C2—C3—H3	120.2
N1^i^—Ni1—O1^i^	94.39 (5)	C5—C4—C3	119.24 (16)
N1—Ni1—O1^i^	170.17 (6)	C5—C4—H4	120.4
O1—Ni1—O1^i^	93.34 (7)	C3—C4—H4	120.4
Ni1—O1—H11	121.0 (15)	N1—C5—C4	121.70 (16)
Ni1—O1—H12	106.4 (18)	N1—C5—C5^i^	114.53 (10)
H11—O1—H12	108 (2)	C4—C5—C5^i^	123.75 (10)
Ni1—O2—H21	109.3 (17)	N1—C7—C2	123.09 (15)
Ni1—O2—H22	119.7 (19)	N1—C7—H7	118.5
H21—O2—H22	109 (2)	C2—C7—H7	118.5
H51—O5—H52	104 (3)		
			
O2^i^—Ni1—N1—C7	−99.70 (14)	C7—C2—C3—C4	1.0 (3)
O2—Ni1—N1—C7	74.51 (14)	C1—C2—C3—C4	−177.65 (16)
N1^i^—Ni1—N1—C7	172.33 (18)	C2—C3—C4—C5	0.2 (3)
O1—Ni1—N1—C7	−14.65 (15)	C7—N1—C5—C4	0.2 (3)
O1^i^—Ni1—N1—C7	127.1 (3)	Ni1—N1—C5—C4	169.99 (14)
O2^i^—Ni1—N1—C5	91.22 (12)	C7—N1—C5—C5^i^	−178.46 (17)
O2—Ni1—N1—C5	−94.57 (12)	Ni1—N1—C5—C5^i^	−8.7 (2)
N1^i^—Ni1—N1—C5	3.25 (9)	C3—C4—C5—N1	−0.9 (3)
O1—Ni1—N1—C5	176.26 (12)	C3—C4—C5—C5^i^	177.6 (2)
O1^i^—Ni1—N1—C5	−42.0 (4)	C5—N1—C7—C2	1.2 (3)
O4—C1—C2—C7	4.6 (3)	Ni1—N1—C7—C2	−167.62 (13)
O3—C1—C2—C7	−173.22 (16)	C3—C2—C7—N1	−1.8 (3)
O4—C1—C2—C3	−176.75 (16)	C1—C2—C7—N1	176.94 (15)
O3—C1—C2—C3	5.4 (3)		

Symmetry codes: (i) −*x*, *y*, −*z*+3/2.

### Hydrogen-bond geometry (Å, °)

**Table:**

*D*—H···*A*	*D*—H	H···*A*	*D*···*A*	*D*—H···*A*
O5—H52···O3^ii^	0.84 (1)	2.18 (2)	2.9566 (19)	155 (3)
O2—H22···O3^iii^	0.83 (1)	1.92 (1)	2.7410 (18)	168 (3)
O5—H51···O4^iv^	0.83 (1)	1.91 (1)	2.7286 (19)	166 (2)
O2—H21···O4^v^	0.83 (1)	1.85 (1)	2.6831 (17)	175 (2)
O1—H11···O4^v^	0.84 (1)	2.65 (2)	3.1740 (18)	122 (2)
O1—H11···O3^v^	0.84 (1)	2.10 (1)	2.9386 (18)	176 (2)
O1—H12···O5	0.83 (1)	1.86 (1)	2.688 (2)	171 (3)

Symmetry codes: (ii) *x*−1/2, −*y*+1/2, *z*−1/2; (iii) *x*, −*y*, *z*−1/2; (iv) −*x*, −*y*+1, −*z*+2; (v) −*x*+1/2, −*y*+1/2, −*z*+2.
